# Concise synthesis of the A/BCD-ring fragment of gambieric acid A

**DOI:** 10.3389/fchem.2014.00116

**Published:** 2015-01-13

**Authors:** Haruhiko Fuwa, Ryo Fukazawa, Makoto Sasaki

**Affiliations:** Graduate School of Life Sciences, Tohoku UniversitySendai, Japan

**Keywords:** marine polycyclic ethers, oxiranyl anions, 6-endo cyclization, oxidative lactonization, palladium-catalyzed reactions

## Abstract

Gambieric acid A (GAA) and its congeners belong to the family of marine polycyclic ether natural products. Their highly complex molecular architecture and unique biological activities have been of intense interest within the synthetic community. We have previously reported the first total synthesis, stereochemical reassignment, and preliminary structure–activity relationships of GAA. Here we disclose a concise synthesis of the A/BCD-ring fragment of GAA. The synthesis started from our previously reported synthetic intermediate that represents the A/B-ring. The C-ring was synthesized via an oxiranyl anion coupling and a 6-*endo* cyclization, and the D-ring was forged by means of an oxidative lactonization and subsequent palladium-catalyzed functionalization of the lactone ring. In this manner, the number of linear synthetic steps required for the construction of the C- and D-rings was reduced from 22 to 11.

## Introduction

**Figure 1:**
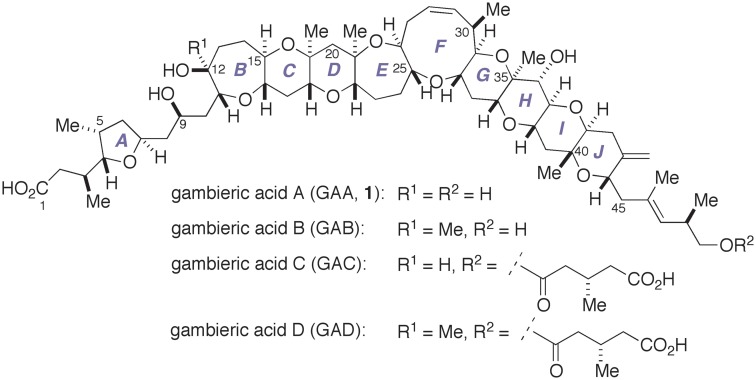
**Structures of gambieric acids A–D**.

In 1992, Nagai, Yasumoto, and co-workers reported the isolation of gambieric acid A (GAA, **1**) and its natural congeners, gambieric acids B–D (GAB–GAD, Figure 1) (Nagai et al., 1992a,b). Gambieric acids (GAs) are the secondary metabolites of the ciguatera causative dinoflagellate *Gambierdiscus toxicus* and belong to the family of marine polycyclic ether natural products (Yasumoto and Murata, 1993; Murata and Yasumoto, 2000). The gross structure and the relative configuration of the polycyclic ether region of GAs were determined on the basis of extensive 2D NMR experiments. The complete stereochemical assignment of GAs was subsequently made through conformational analysis of GAB on the basis of nuclear Overhauser effect (NOE) correlations coupled with ^3^*J*_H,H_ values, application of chiral anisotropic reagents, and chiral HPLC analysis of degradation products (Morohashi et al., 2000). However, our synthesis and NMR spectroscopic analysis of a series of suitably designed A/B-ring model compounds of GAs strongly indicated that the absolute configuration of the polycyclic ether domain of GAs needs to be unambiguously established through total synthesis (Fuwa et al., 2008a, 2009a). The *trans*-fused polycyclic ether backbone of GAs is the common structural characteristic shared among the family of marine polycyclic ether neurotoxins, e.g., brevetoxins, ciguatoxins, and gambierol. Nonetheless, it has been reported that GAA shows only moderate toxicity against mice or cultured mammalian cells (Nagai et al., 1992b) and only weakly displaces binding of tritiated dihydrobrevetoxin B ([^3^H]-PbTx-3) to voltage-gated sodium channels (Inoue et al., 2003). Instead, GAs are known to impart extraordinary potent antifungal activity against *Aspergillus niger*, which is approximately 2000 times greater than that of amphotericin B (Nagai et al., 1993). In addition, it has been described that GAA is a possible endogenous growth-regulating factor of *G. toxicus* (Sakamoto et al., 1996). Unfortunately, the molecular basis for the biological activities of GAs has not been elucidated at all, partly due to the natural scarcity of these substances. The molecular complexity and intriguing biological activities of GAs have attracted the attention of the synthetic community (Kadota et al., 2001a,b; Clark et al., 2004, 2005; Sato and Sasaki, 2005, 2007; Fuwa et al., 2007, 2008a, 2009a,b, 2010; Roberts and Rainier, 2007; Saito and Nakata, 2009; Tsubone et al., 2011a,b).

**Figure 2:**
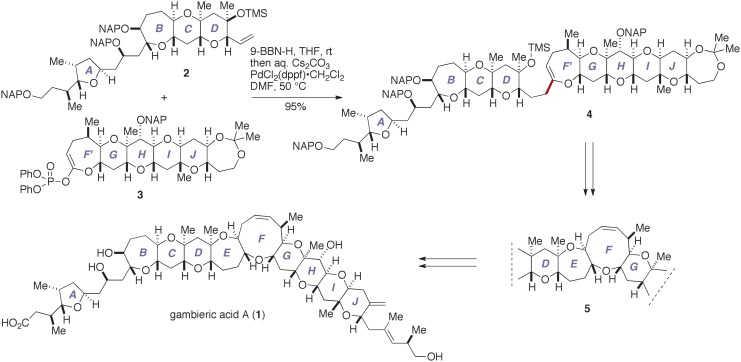
**Outline of our total synthesis of gambieric acid A**. aq., aqueous; 9-BBN-H, 9-borabicyclo[3.3.1]nonane; DMF, *N*,*N*-dimethylformamide; dppf, 1,1′-bis(diphenylphosphino)ferrocene; NAP, 2-naphthylmethyl; rt, room temperature; THF, tetrahydrofuran; TMS, trimethylsilyl.

We have recently completed the first total synthesis of GAA to establish its absolute configuration as that shown by **1** (Fuwa et al., 2012; Ishigai et al., 2013; Sasaki and Fuwa, 2014). Our synthesis entailed convergent assembly of the A/BCD- and F′GHIJ-ring fragments, i.e., **2** and **3**, respectively, by means of Suzuki–Miyaura coupling (Miyaura and Suzuki, 1995; Sasaki and Fuwa, 2008; Suzuki, 2011) to give the endocyclic enol ether **4**, followed by closure of the E- and F-rings via a stereoselective allylation of a thioacetal (Suga et al., 2014) and a ring-closing metathesis (Hoveyda and Zhugralin, 2007), respectively, to construct the nonacyclic polyether core **5** (Figure 2). Moreover, we have prepared several synthetic analogs of GAA by diversifying the synthetic route from the nonacyclic ether **5** and investigated the structure–activity relationships (SARs) of the peripheral substituents on the polycyclic ether skeleton (Ishigai et al., 2013). Toward the elucidation of the SARs of GAA in greater detail, however, it deemed indispensable to improve the synthetic availability of **2** and **3**. Here we describe a concise synthesis of the A/BCD-ring fragment **2** of GAA, wherein the C-ring was constructed by using an oxiranyl anion coupling/6-*endo* cyclization sequence (Mori et al., 1997a,b, 1998) and the D-ring was forged via an oxidative lactonization and subsequent palladium-catalyzed functionalization of the derived lactone.

## Materials and methods

Detailed experimental procedure and compound characterization data are furnished in the Supplementary Material.

## Results and discussion

**Figure 3:**
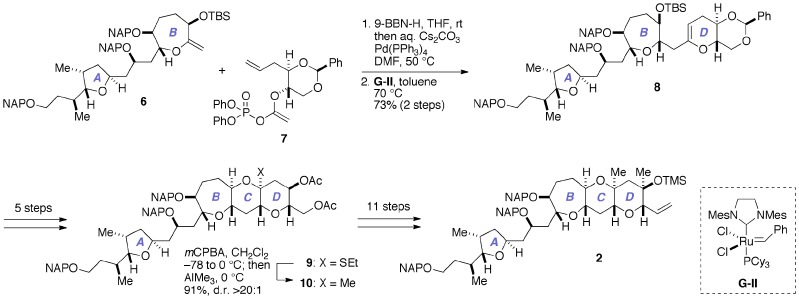
**Outline of our previous synthesis of the A/BCD-ring fragment 2 of gambieric acid A**. Cy, cyclohexyl; *m*CPBA, *m*-chloroperoxybenzoic acid; d.r., diastereomer ratio; Mes, 2,4,6-trimethylphenyl (mesityl); TBS, *t*-butyldimethylsilyl.

As delineated in Figure 3, our previous synthesis of **2** (Fuwa et al., 2012; Ishigai et al., 2013) relied upon Suzuki–Miyaura coupling of an alkylborane prepared *in situ* from the A/B-ring exocyclic enol ether **6** with the enol phosphate **7**, followed by ring-closing metathesis of the derived enol ether (Fuwa and Sasaki, 2008b). The closure of the C-ring was achieved by means of stereoselective methylation of the thioacetal **9** (Nicolaou et al., 1989; Fuwa et al., 2001), and subsequent elaboration of the D-ring completed the synthesis of **2**. Although sufficient quantities of **2** for the total synthesis could actually be prepared, the synthetic sequence from **6** to **2** was rather lengthy (19 steps), partly because multiple steps were required for the introduction of the 1,3-diaxial methyl groups onto the D-ring.

**Figure 4:**
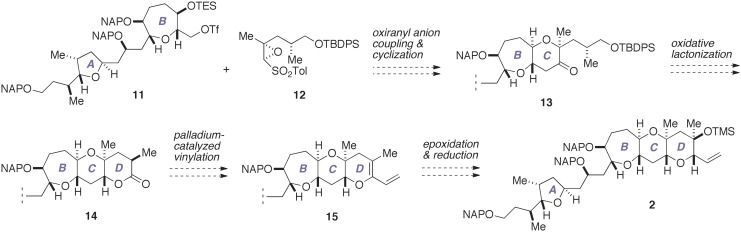
**Improved synthesis plan toward 2**. TBDPS, *t*-butyldiphenylsilyl; TES, triethylsilyl; Tf, trifluoromethanesulfonate (triflate); Tol, *p*-tolyl.

With our previous synthesis in mind, we devised an improved synthesis of **2**, which is outlined in Figure 4. Currently, a number of synthetic methods are available for the synthesis of tetrahydropyran derivatives (Nasir et al., 2014). We envisioned that the C-ring could be efficiently constructed in a concise manner by exploiting the chemistry developed by Mori et al. (1997a,b, 1998). Thus, a coupling of the triflate **11**, which represents the A/B-ring, with an oxiranyl anion generated from the epoxy sulfone **12**, followed by acid-catalyzed cleavage of the silyl ether and spontaneous 6-*endo* cyclization would directly afford the A/BC-ring tricycle **13**. Meanwhile, the oxiranyl anion chemistry cannot be directly applied to the D-ring with 1,3-diaxial methyl groups. Accordingly, we planned to construct the D-ring via the lactone **14**. Functionalization of lactones is a versatile means for the synthesis of cyclic ethers (e.g., Nicolaou et al., 1997; Suga et al., 2014). A palladium-catalyzed vinylation of an enol phosphate or triflate derived from **14** would give the diene **15**. Chemo- and stereoselective epoxidation of **15** and subsequent stereoselective reduction of the resultant epoxide would allow a rapid access to the targeted **2**.

**Figure 5:**
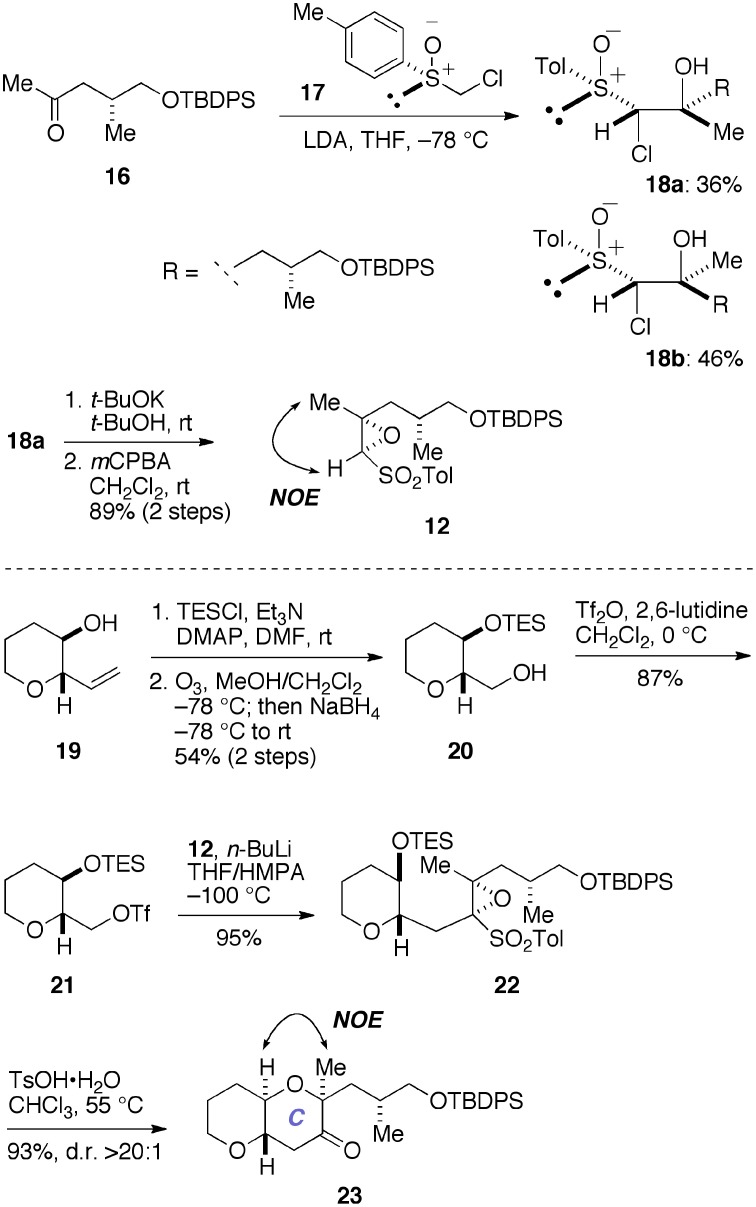
**Synthesis of epoxy sulfone 12 and a model study for the construction of the C-ring**. DMAP, 4-(dimethylamino)pyridine; HMPA, hexamethylphosphoramide; LDA, lithium diisopropylamide; NOE, nuclear Overhauser effect; TsOH, *p*-toluenesulfonic acid.

Initially, we prepared the epoxy sulfone **12** and examined its use in a model system (Figure 5). The synthesis of **12** started with the known methyl ketone **16** (Edmunds et al., 1997). Coupling of **16** with a lithiated sulfoxide generated *in situ* from **17** (Satoh et al., 1989; Mori et al., 1998) provided the chlorohydrins **18a** (36%) and **18b** (46%) as a separable mixture. The minor diastereomer **18a** was treated with a base and then oxidized with *m*CPBA to afford the epoxy sulfone **12** (89%, two steps). At this stage, however, we were unable to establish the absolute configuration of the newly introduced stereogenic centers of **12**. Accordingly, we reacted an oxiranyl anion prepared from **12** with the triflate **21** as a model experiment. The triflate **21** was readily prepared from the known alcohol **19** (Inoue et al., 1999) in three steps, including silylation, ozonolysis/NaBH_4_ reduction, and triflation. Treatment of a mixture of **12** and **21** with *n*-BuLi in THF/HMPA at −100°C cleanly provided the desired coupling product **22** (95%). Exposure of **22** to TsOH·H_2_O in CHCl_3_ at 55°C resulted in cleavage of the TES ether and spontaneous 6-*endo* cyclization, as expected, to afford the ketone **23** in 93% yield as a single stereoisomer (d.r. >20:1). Here we were able to establish the stereostructure of **23** by an NOE experiment as shown, thus confirmed the absolute configuration of the epoxy sulfone **12**.

**Figure 6:**
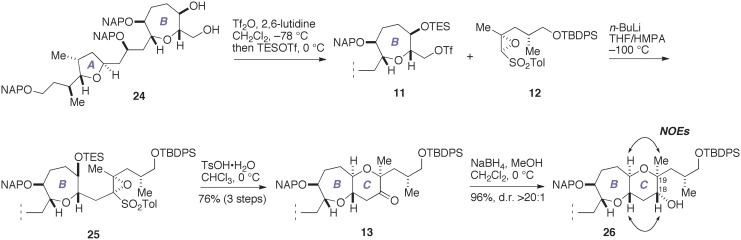
**Construction of the C-ring**.

With the requisite epoxy sulfone **12** available, we proceeded to construct the C-ring in the real system, as shown in Figure 6. Sequential triflation/silylation (Mori et al., 1997a) of the AB-ring diol **24** (Fuwa et al., 2012; Ishigai et al., 2013) gave the triflate **11**. This was immediately coupled with an oxiranyl anion generated from **12** under the same conditions employed above (*n*-BuLi, THF/HMPA, −100°C) to afford the coupling product **25**. Subsequent treatment of **25** with TsOH·H_2_O in CHCl_3_ at 0°C led to the ketone **13** in 76% overall yield from **24**. Stereoselective reduction of **13** with NaBH_4_ afforded the alcohol **26** (96%, d.r. >20:1). The absolute configuration of the C18 and C19 stereogenic centers was confirmed by NOE experiments, as shown. Thus, we successfully elaborated the C-ring in only four steps from **24**.

**Figure 7:**
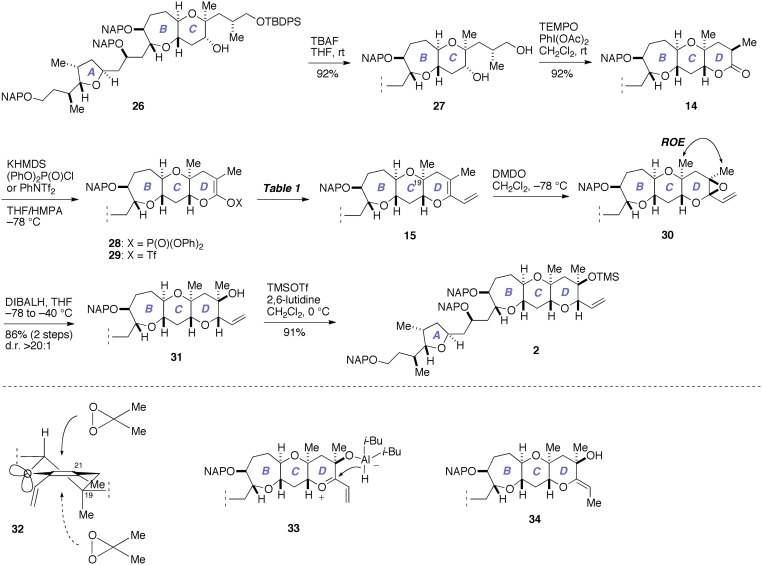
**Construction of the D-ring and completion of the synthesis of 2**. DIBALH, diisobutylaluminum hydride; DMDO, dimethyldioxirane; KHMDS, potassium bis(trimethylsilyl)amide; ROE, rotating-frame Overhauser effect; TBAF, tetra-*n*-butylammonium fluoride; TEMPO, 2,2,6,6-tetramethylpiperidin-1-oxyl.

Next, we investigated the construction of the D-ring, as shown in Figure 7. Removal of the silyl group from **26** with TBAF gave the diol **27** (92%), which was oxidized with TEMPO/PhI(OAc)_2_ (Hansen et al., 2003) to directly afford the lactone **14** (92%).

We investigated the functionalization of the lactone ring of **14** to elaborate the D-ring. Exposure of **14** to KHMDS in the presence of (PhO)_2_P(O)Cl smoothly provided the enol phosphate **28** (Nicolaou et al., 1997). Initially, we examined the palladium-catalyzed vinylation of **28** under Suzuki–Miyaura conditions (Miyaura and Suzuki, 1995; Suzuki, 2011), as summarized in Table 1. Treatment of **28** with vinylboronic acid pinacol ester under the influence of aqueous Cs_2_CO_3_ solution and PdCl_2_(dppf)·CH_2_Cl_2_ catalyst, however, did not give the diene **15** at all and only returned the enol phosphate **28** (entry 1). Changing the catalyst to Pd(PPh_3_)_4_ was also ineffective (entry 2). We suspected that the low reactivity of the enol phosphate **28** would stem from the steric bulk of the α-methyl group (e.g., Nicolaou et al., 1997). Thus, we also prepared the enol triflate **29** (Tsushima et al., 1989) as a more reactive surrogate. Because our previous studies have shown that highly reactive enol triflates favor palladium catalyst with electron deficient supporting ligands (Sasaki et al., 1998, 2002), we examined Suzuki–Miyaura coupling of **29** with vinylboronic acid pinacol ester under the influence of the Pd_2_(dba)_3_/Ph_3_As catalyst system (entries 3 and 4). To our dismay, we isolated **15** in only moderate yields under these conditions. These unsatisfactory results could be ascribed to undesirable hydrolysis of **29** under alkaline conditions. Accordingly, we turned our attention to Stille coupling of **29** with vinyl(tri-*n*-butyl)stannane by the action of Pd(PPh_3_)_4_ catalyst and LiCl in 1,4-dioxane at 80°C (Scott and Stille, 1986) (entry 5). Under these conditions, we were able to isolate the diene **15** in 63% overall yield from **14**. Here it was necessary to purify the diene **15** by aqueous 20% KF and DL-serine workup and by flash column chromatography using potassium carbonate–silica gel to scavenge organotin byproducts and palladium salts (Leibner and Jacobus, 1979; Harrowven et al., 2010; Yoshimura et al., 2011), as traces of these weakly Lewis acidic contaminants were found to adversely affect the outcome of subsequent epoxidation process.

**Table 1:** **Examination of palladium-catalyzed vinylation of enol phosphate 28 and triflate 29**.

**Entry**	**Substrate**	**Reagents and conditions**	**Yield (from 14) (%)**
1	**28**	vinylBpin, aq. Cs_2_CO_3_, PdCl_2_(dppf)·CH_2_Cl_2_, DMF, 50°C	0
2	**28**	vinylBpin, aq. Cs_2_CO_3_, Pd(PPh_3_)_4_, DMF, 50°C	0
3	**29**	vinylBpin, aq. Cs_2_CO_3_, Pd_2_(dba)_3_, Ph_3_As, DMF, rt	39
4	**29**	vinylBpin, aq. NaHCO_3_, Pd_2_(dba)_3_, Ph_3_As, DMF, rt	20
5	**29**	vinylSnBu_3_, Pd(PPh_3_)_4_, LiCl, 1,4-dioxane, 80°C	63

*dba, dibenzylideneacetone; pin, pinacolate*.

Our final task was to elaborate the diene **15** to the A/BCD-ring fragment **2** via chemo- and stereoselective epoxidation of **15** and subsequent reductive opening of the derived epoxide **30** (Figure 7). Thus, treatment of **15** with DMDO in CH_2_Cl_2_ at −78°C provided the epoxide **30** as a single stereoisomer (d.r. >20:1, judged by ^1^H NMR analysis). This epoxide was isolated by aqueous workup and immediately reduced with DIBALH in THF at −78 to −40°C to afford the tertiary alcohol **31** in 86% yield (two steps). The chemoselectivity of the epoxidation of **15** was secured by the differential reactivity of the enol ether and the terminal olefin (Fujiwara et al., 1999; Clark et al., 2007). The stereochemical outcome of the epoxidation of **15** with DMDO was in accordance with that of glycal derivatives (Halcomb and Danishefsky, 1989; Allwein et al., 2002) and could be reasoned by considering stereoelectronic effect as well as the steric bulk of the axial methyl group at the C19 position (e.g., **32**). The purity of the diene **15** was crucial for the success of the epoxidation; when **15** containing traces of organotin byproducts and/or palladium salts was used, *in situ* hydrolysis of the epoxide **30** with traces of adventitious H_2_O occurred as a serious side reaction. Meanwhile, the stereoselectivity of the DIBALH reduction of the epoxide **30** could be explained by considering the aluminum ate complex **33** as the intermediate, as previously proposed by Majumder et al. (2006). Our initial attempts to reduce **30** with DIBALH in CH_2_Cl_2_ at −78°C resulted in only 19% yield of the tertiary alcohol **31** and the exocyclic enol ether **34** was isolated alongside in 44% yield. The undesired product **34** might arise from an S_N_2′-type reduction of **33**. Consequently, we chose to perform the reduction in THF to reduce the Lewis acidity of DIBALH as well as to solvate the presumed oxocarbenium ion intermediate **33**. Other reducing conditions, such as Et_3_SiH/BF_3_·OEt_2_ (Clark et al., 2007) or NaBH_3_CN (Zimmermann et al., 2000), gave unsatisfactory results. Finally, silylation of **31** with TMSOTf/2,6-lutidine afforded the A/BCD-ring fragment **2** in 91% yield.

## Conclusions

In this paper, we described a concise synthesis of the A/BCD-ring fragment **2** of GAA, which is significantly improved over our previous synthesis in terms of “step economy” (Wender et al., 2008). Starting from the A/B-ring diol **24**, the C-ring was rapidly constructed by means of an oxiranyl anion coupling and subsequent 6-*endo* cyclization. The D-ring was first forged as a six-membered lactone and further elaborated via a Stille coupling. The present synthesis minimized the use of protecting group chemistry and enabled rapid synthesis of **2** from **24** in just 11 linear steps, which compares favorably with our previously reported synthesis (22 linear steps from **24**).

### Conflict of interest statement

The authors declare that the research was conducted in the absence of any commercial or financial relationships that could be construed as a potential conflict of interest.
