# Supplementary material for: Concise synthesis of the A/BCD-ring fragment of gambieric acid A
Source: Front Chem. 2015 Jan 13;2:116. doi: 10.3389/fchem.2014.00116 (PMC4292782; doi:10.3389/fchem.2014.00116)
Supplement: Supplementary file 1 [file DataSheet1.DOC]

***Supplementary Material***

**Concise synthesis of the A/BCD-ring fragment of gambieric acid A**

**Haruhiko Fuwa^1^*, Ryo Fukazawa^1^, and Makoto Sasaki^1^**

^1^Graduate School of Life Sciences, Tohoku University, Sendai, Japan

*** Correspondence:** Haruhiko Fuwa, Graduate School of Life Sciences, Tohoku University, 2-1-1 Katahira, Aoba-ku, Sendai 980-8577, Japan.

hfuwa@m.tohoku.ac.jp

1. **Supplementary Data**

Experimental details and spectroscopic data for new compounds are provided in this Supplementary Material.

**General remarks.** All reactions sensitive to moisture and/or air were carried out under an atmosphere of argon in dry, freshly distilled solvents under anhydrous conditions using oven-dried glassware unless otherwise noted. Anhydrous dichloromethane (CH_2_Cl_2_) was purchased from Kanto Chemical Co. Inc. and used directly without further drying. Anhydrous tetrahydrofuran (THF) was purchased from Wako Pure Chemical Industries, Ltd. and further purified by a Glass Contour solvent purification system under an atmosphere of argon immediately prior to use. Diisopropylamine (*i*-Pr_2_NH), diisopropylethylamine (*i*-Pr_2_NEt), 2,6-lutidine, and triethylamine (Et_3_N) were distilled from calcium hydride under an atmosphere of argon. 1,4-Dioxane was distilled from sodium benzophenone ketyl under an atmosphere of argon. Hexamethylphosphoramide (HMPA) was distilled from calcium hydride under reduced pressure. All other chemicals were purchased at highest commercial grade and used directly. Analytical thin-layer chromatography (TLC) was performed using E. Merck silica gel 60 F_254_ plates (0.25-mm thickness). Flash column chromatography was carried out using Fuji Silysia silica gel BW-300 (200–400 mesh). Optical rotations were recorded on a JASCO P-1020 digital polarimeter. IR spectra were recorded on a JASCO FT/IR-4100 spectrometer. ^1^H and ^13^C NMR spectra were recorded on a JEOL JNM ECA-600 spectrometer, and chemical shift values are reported in ppm (δ) downfield from tetramethylsilane with reference to internal residual solvent [^1^H NMR, CHCl_3_ (7.24), C_6_HD_5_ (7.15); ^13^C NMR, CDCl_3_ (77.0), C_6_D_6_ (128.0)]. Coupling constants (*J*) are reported in Hertz (Hz). The following abbreviations were used to designate the multiplicities: s = singlet; d = doublet; t = triplet; q = quartet; m = multiplet; br = broad. ESI-TOF mass spectra were measured on a Bruker microTOFfocus spectrometer. Diastereomer ratio (d.r.) was estimated by ^1^H NMR spectroscopic analysis (600 MHz), unless otherwise noted.

**Chlorohydrins 18a and 18b.** To a solution of *i*-Pr_2_NH (0.050 mL, 0.35 mmol) in THF (0.80 mL) at 0 °C was added *n*-BuLi (1.58 M solution in *n*-hexane, 0.20 mL, 0.32 mmol), and the resultant solution was stirred at 0 °C for 30 min. To the solution at −78 °C was added a solution of sulfoxide **17** (58.9 mg, 0.312 mmol) in THF (0.6 mL + 0.6 mL rinse), and the resultant solution was stirred at −78 °C for 30 min. To the solution was added a solution of methyl ketone **16** (132 mg, 0.373 mmol) in THF (0.6 mL + 0.6 mL rinse), and the resultant solution was stirred at −78 °C for 1 h. The reaction was quenched with saturated aqueous Na_2_SO_3_ solution. The resultant mixture was allowed to warm to room temperature and then extracted with EtOAc. The organic layer was washed with H_2_O and brine, dried (Na_2_SO_4_), filtered, and concentrated under reduced pressure. Purification of the residue by flash column chromatography (silica gel, 10−30% EtOAc/hexanes) gave chlorohydrin **18a** (60.6 mg, 36%) along with diastereomeric chlorohydrin **18b** (77.7 mg, 46%) as colorless oils. Data for **18a**: [α]_D_^24^ +102.1 (*c* 1.00, CHCl_3_); IR (film) 3734, 3648, 3365, 2929, 2360, 1652, 1472, 1427, 1111, 740 cm^−1^; ^1^H NMR (600 MHz, CDCl_3_) δ 7.72–7.69 (m, 4H), 7.51 (d, *J* = 8.2 Hz, 2H), 7.45–7.39 (m, 6H), 7.34–7.30 (m, 2H), 5.42 (s, 1H), 4.26 (s, 1H), 3.59 (dd, *J* = 10.1, 4.1 Hz, 1H), 3.40 (dd, *J* = 9.7, 9.6 Hz, 1H), 2.40 (s, 3H), 2.25 (dd, *J* = 14.7, 7.4 Hz, 1H), 2.10 (m, 1H), 1.91 (dd, *J* = 14.7, 2.8 Hz, 1H), 1.44 (s, 3H), 1.08 (s, 9H), 0.84 (d, *J* = 6.9 Hz, 3H); ^13^C NMR (150 MHz, CDCl_3_) δ 141.6, 139.3, 135.7 (2C), 135.6 (2C), 132.4, 132.2, 130.0 (2C), 129.7 (2C), 127.92 (2C), 127.87 (2C), 124.5 (2C), 88.6, 75.4, 70.8, 41.7, 30.8, 26.8 (3C), 25.9, 21.4, 19.3, 19.1; HRMS (ESI) calcd for C_30_H_39_ClO_3_SSiNa [(M + Na)^+^] 565.1970, found 565.1966. Data for **18b**: [α]_D_^25^ +115.4 (*c* 1.00, CHCl_3_); IR (film) 3853, 3648, 3357, 2360, 1653, 1492, 1389, 1015, 940, 740 cm^−1^; ^1^H NMR (600 MHz, CDCl_3_) δ 7.64–7.60 (m, 4H), 7.46–7.37 (m, 6H), 7.29 (d, *J* = 8.3 Hz, 2H), 7.20 (d, *J* = 7.8 Hz, 2H), 5.29 (br s, 1H), 4.28 (s, 1H), 3.56 (dd, *J* = 10.6, 3.7 Hz, 1H), 3.37 (dd, *J* = 10.1, 9.6 Hz, 1H), 2.42 (s, 3H), 2.02 (dd, *J* = 15.1, 2.8 Hz, 1H), 1.88 (m, 1H), 1.72 (dd, *J* = 15.1, 8.2 Hz, 1H), 1.52 (s, 3H), 1.10 (s, 9H), 0.77 (d, *J* = 6.8 Hz, 3H); ^13^C NMR (150 MHz, CDCl_3_) δ 141.4, 139.2, 135.4 (2C), 135.3 (2C), 132.5, 132.0, 130.0 (2C), 129.7 (2C), 127.92 (4C), 124.4 (2C), 85.9, 75.1, 70.9, 47.3, 31.2, 26.9 (3C), 24.1, 21.3, 19.1, 18.8; HRMS (ESI) calcd for C_30_H_39_ClO_3_SSiNa [(M + Na)^+^] 565.1970, found 565.1994.

**Epoxy sulfone 12.** To a solution of chlorohydrin **18a** (319 mg, 0.589 mmol) in *t-*BuOH (10 mL) at room temperature was added *t*-BuOK (80.1 mg, 0.714 mmol), and the resultant mixture was stirred at room temperature for 1 h. The reaction mixture was diluted with EtOAc, washed with H_2_O and brine, dried (Na_2_SO_4_), filtered, and concentrated under reduced pressure. The residue was roughly purified by flash column chromatography (silica gel, 15% EtOAc/hexanes) to give crude epoxide, which was used in the next reaction without further purification.

To a solution of the above epoxide in CH_2_Cl_2_ (10 mL) at room temperature was added *m*CPBA (142 mg, 0.823 mmol), and the resultant mixture was stirred at room temperature for 7.5 h. The reaction was quenched with saturated aqueous Na_2_SO_3_ solution. The resultant mixture was extracted with EtOAc, and the organic layer was washed with saturated aqueous NaHCO_3_ solution and brine, dried (Na_2_SO_4_), filtered, and concentrated under reduced pressure. Purification of the residue by flash column chromatography (silica gel, 10% EtOAc/hexanes) gave epoxy sulfone **12** (275.5 mg, 89% for the two steps) as a colorless oil: [α]_D_^27^ −75.6 (*c* 1.00, benzene); IR (film) 3853, 3734, 2361, 1716, 1558, 1540, 1507, 1153, 1109, 701 cm^−1^; ^1^H NMR (600 MHz, C_6_D_6_) δ 7.85–7.79 (m, 6H), 7.25–7.18 (m, 6H), 6.70 (d, *J* = 7.8 Hz, 2H), 3.69 (dd, *J* = 10.1, 5.9 Hz, 1H), 3.62 (dd, *J* = 10.1, 6.4 Hz, 1H), 3.45 (s, 1H), 2.49 (dd, *J* = 14.2, 6.4 Hz, 1H), 2.39 (dd, *J* = 14.2, 8.3 Hz, 1H), 1.94 (m, 1H), 1.81 (s, 3H), 1.20 (s, 9H), 1.19 (d, *J* = 6.4 Hz, 3H), 0.89 (s, 3H); ^13^C NMR (150 MHz, C_6_D_6_) δ 144.6, 137.4, 136.09 (2C), 136.07 (2C), 134.31, 134.28, 129.9 (2C), 128.7 (2C), 128.3 (3C), 127.8 (3C), 74.7, 69.0, 67.2, 34.8, 33.9, 27.2 (3C), 22.7, 21.1, 19.6, 17.2; HRMS (ESI) calcd for C_30_H_38_O_4_SSiNa [(M + Na)^+^] 545.2152, found 545.2164.

**Alcohol 20.** To a solution of alcohol **19** (159 mg, 1.23 mmol), Et_3_N (0.55 mL, 4.0 mmol), and DMAP (15.1 mg, 0.123 mmol) in DMF (10 mL) at 0 °C was added TESCl (0.25 mL, 1.5 mmol), and the resultant solution was stirred at room temperature for 2 h. The reaction mixture was diluted with *t*-BuOMe, washed with H_2_O and brine, dried (Na_2_SO_4_), filtered, and concentrated under reduced pressure. The residue was roughly purified by flash column chromatography (silica gel, 20% EtOAc/hexanes) gave crude silyl ether (185 mg), which was used in the next reaction without further purification.

Ozone was bubbled through a solution of the above silyl ether (185 mg) in MeOH/CH_2_Cl_2_ (1:1, v/v, 6 mL) at −78 °C for 40 min. Excess ozone was removed by oxygen purge. To the reaction mixture at −78 °C was added NaBH_4_ (116 mg, 3.07 mmol), and the resultant solution was allowed to warm to room temperature overnight. The reaction mixture was diluted with EtOAc, washed with H_2_O and brine, dried (Na_2_SO_4_), filtered, and concentrated under reduced pressure. Purification of the residue by flash column chromatography (silica gel, 5−10% EtOAc/hexanes) gave alcohol **20** (165.0 mg, 54% for the two steps) as a colorless oil: [α]_D_^25^ −55.3 (*c* 1.00, CHCl_3_); IR (film) 3420, 2930, 2361, 1671, 1508, 1373, 1073, 1081, 752 cm^−1^; ^1^H NMR (600 MHz, CDCl_3_) δ 3.89 (m, 1H), 3.81 (dd, *J* = 11.5, 3.2 Hz, 1H), 3.60 (d, *J* =11.5, 6.0 Hz, 2H), 3.48 (ddd, *J* = 11.4, 10.6, 4.6 Hz, 1H), 3.34 (m, 1H), 3.13 (ddd, *J* = 6.0, 3.2, 3.2 Hz, 1H), 2.01 (m, 1H), 1.64–1.42 (m, 3H), 0.94 (t, *J* = 7.8 Hz, 9H), 0.84 (q, *J* = 7.8 Hz, 6H); ^13^C NMR (150 MHz, CDCl_3_) δ 82.3, 67.9, 67.6, 63.1, 33.3, 25.4, 6.7 (3C), 4.9 (3C); HRMS calcd for C_12_H_26_O_3_SiNa [(M + Na)^+^] 269.1543, found 269.1566.

**Triflate 21.** To a solution of alcohol **20** (23.7 mg, 0.0962 mmol) and 2,6-lutidine (0.040 mL, 0.34 mmol) in CH_2_Cl_2_ (1.0 mL) at 0 °C was added Tf_2_O (0.020 mL, 0.12 mmol), and the resultant solution was stirred at 0 °C for 1 h. The reaction mixture was diluted with EtOAc, washed with saturated aqueous NaHCO_3_ solution and brine, dried (Na_2_SO_4_), filtered, and concentrated under reduced pressure. Purification of the residue by flash column chromatography (silica gel, 5% EtOAc/hexanes) gave triflate **21** (31.8 mg, 87%) as a colorless oil: [α]_D_^22^ −56.8 (*c* 0.30, CHCl_3_); IR (film) 3853, 3734, 2925, 2360, 1716, 1653, 1558, 1416, 11146, 1077, 947, 772 cm^−1^; ^1^H NMR (600 MHz, C_6_D_6_) δ 4.56 (dd, *J* = 10.1, 1.9 Hz, 1H), 4.45 (dd, *J* = 10.1, 5.0 Hz, 1H), 3.54 (m, 1H), 3.34 (ddd, *J* =10.1, 9.1, 4.6 Hz, 2H), 2.93 (ddd, *J* = 5.0, 4.6, 1.8 Hz, 1H), 2.82 (ddd, *J* = 12.4, 11.5, 2.3 Hz, 1H), 1.70 (m, 1H), 1.25–1.01 (m, 2H), 0.91 (t, *J* = 7.8 Hz, 9H), 0.47 (q, *J* = 7.8 Hz, 6H); ^13^C NMR (150 MHz, CDCl_3_) δ 172.3, 79.8, 76.1, 67.8, 66.8, 33.3, 25.0, 6.7 (3C), 5.0 (3C); HRMS (ESI) calcd for C_13_H_25_O_5_F_3_SSiNa [(M + Na)^+^] 401.1036, found 401.1036.

**Coupling product 22.** To a solution of epoxy sulfone **12** (11.4 mg, 0.0218 mmol) and triflate **21** (13.6 mg, 0.0359 mmol) in THF/HMPA (20:1, v/v, 1.05 mL) at −100 °C was added dropwise *n*-BuLi (1.58 M solution in *n*-hexane, 0.015 mL, 0.024 mmol), and the resultant solution was stirred at −100 °C for 1 h. The reaction was quenched with saturated aqueous NH_4_Cl solution. The resultant mixture was allowed to warm to room temperature and then extracted with EtOAc. The organic layer was washed with brine, dried (Na_2_SO_4_), filtered, and concentrated under reduced pressure. Purification of the residue by flash column chromatography (silica gel, 5% EtOAc/hexanes) gave coupling product **22** (15.4 mg, 95%) as a colorless oil: [α]_D_^23^ −30.4 (*c* 1.00, CHCl_3_); IR (film) 3853, 3734, 3566, 2954, 2360, 1652, 1558, 1322, 1152, 1097, 812 cm^−1^; ^1^H NMR (600 MHz, C_6_D_6_) δ 8.40 (d, *J* = 8.3 Hz, 2H), 7.82–7.80 (m, 4H), 7.25–7.22 (m, 6H), 6.82 (d, *J* = 8.3 Hz, 2H), 3.78 (dd, *J* = 10.1, 5.9 Hz, 1H), 3.63 (dd *J* = 13.3, 5.1 Hz, 1H), 3.61 (dd, *J* = 10.1, 5.9 Hz, 1H), 3.07 (ddd, *J* = 8.8, 8.7, 4.6 Hz, 1H), 2.94 (dd, *J* = 11.9, 11.5 Hz, 1H), 2.90 (d, *J* = 16.0 Hz, 1H), 2.83 (dd, *J* = 9.7, 8.7 Hz, 1H), 2.67 (dd, *J* = 13.8, 5.5 Hz, 1H), 2.56 (dd, *J* = 13.8, 8.7 Hz, 1H), 2.20–2.16 (m, 2H), 1.86 (s, 3H), 1.70 (m, 1H), 1.43 (s, 3H), 1.35–1.29 (m, 4H), 1.26 (d, *J* = 6.4 Hz, 3H), 1.12–0.95 (m, 6H), 0.94 (t, *J* = 7.8 Hz, 9H), 0.52 (q, *J* = 7.8 Hz, 6H); ^13^C NMR (150 MHz, C_6_D_6_) δ 143.8, 137.7, 136.11 (2C), 136.08 (2C), 134.49, 134.41, 130.2 (2C), 129.8 (2C), 129.4 (2C), 128.0 (2C), 127.8 (2C), 80.6 (2C), 71.6, 69.3, 69.1, 67.4, 36.3, 34.0, 33.9, 31.3, 27.2 (3C), 25.7, 22.1, 21.1, 19.6, 17.3, 7.1 (3C), 5.3 (3C); HRMS (ESI) calcd for C_42_H_62_O_6_SSi_2_Na [(M + Na)^+^] 773.3698, found 773.3692.

**Ketone 23.** To a solution of coupling product **22** (6.7 mg, 8.9 μmol) in CHCl_3_ (0.2 mL) at room temperature was added TsOH·H_2_O (3.1 mg, 0.016 mmol), and the resultant solution was stirred at 55 °C for 1 h. The reaction mixture was neutralized with Et_3_N, diluted with EtOAc, washed with brine, dried (Na_2_SO_4_), filtered, and concentrated under reduced pressure. Purification of the residue by flash column chromatography (silica gel, 2−5% EtOAc/hexanes) gave ketone **23** (4.0 mg, 93%) as a colorless oil: [α]_D_^23^ +26.7 (*c* 0.30, CHCl_3_); IR (film) 3853, 3839, 2928, 2370, 2324, 2370, 1698, 1488, 1418 cm^−1^; ^1^H NMR (600 MHz, CDCl_3_) δ 7.81–7.78 (m, 4H), 7.24–7.21 (m, 6H), 3.81 (dd, *J* = 10.1, 5.5 Hz, 1H), 3.58 (dd, *J* = 11.0, 4.6 Hz, 1H), 3.47 (dd, *J* = 9.6, 9.6 Hz, 1H), 3.13 (ddd, *J* = 9.2, 9.1, 4.6 Hz, 1H), 2.93–2.85 (m, 2H), 2.74 (dd, *J* = 17.0, 5.5 Hz, 1H), 2.33 (dd, *J* = 16.9, 16.5 Hz, 1H), 2.02 (m, 1H), 1.91 (dd, *J* = 14.2, 5.9 Hz, 1H), 1.72 (m, 1H), 1.61 (dd, *J* = 14.2, 5.9 Hz, 1H), 1.34 (m, 1H), 1.18 (s, 9H), 1.14 (m, 1H), 1.12 (d, *J* = 6.8 Hz, 3H), 1.04 (m, 1H), 0.99 (s, 3H); ^13^C NMR (150 MHz, CDCl_3_) δ 210.4, 135.62 (2C), 135.60 (2C), 134.2, 134.1, 129.7, 129.5, 129.4, 127.5 (3C), 83.9, 76.1, 70.2, 69.3, 67.3, 43.0, 41.6, 32.3, 29.3, 26.9 (3C), 25.2, 23.1, 19.3, 18.7; HRMS (ESI) calcd for C_29_H_40_O_4_SSiNa [(M + Na)^+^] 503.2588, found 503.2586.

**Ketone 13.** To a solution of diol **24** (1.32 g, 1.66 mmol) and 2,6-lutidine (1.0 mL, 8.6 mmol) in CH_2_Cl_2_ (17 mL) at −78 °C was added Tf_2_O (0.29 mL, 1.7 mmol), and the resultant solution was stirred at −78 °C for 20 min. To the solution was added TESOTf (0.95 mL, 4.2 mmol), and the resultant solution was allowed to warm to 0 °C over a period of 30 min and further stirred at 0 °C for 10 min. The reaction was quenched with saturated aqueous NH_4_Cl solution. The resultant mixture was extracted with EtOAc, and the organic layer was washed with saturated aqueous NaHCO_3_ solution and brine, dried (Na_2_SO_4_), filtered, and concentrated under reduced pressure to give crude triflate **11**, which was immediately used in the next reaction without purification.

To a solution of the above triflate **11** and epoxy sulfone **12** (0.88 g, 1.7 mmol) in THF/HMPA (20:1, v/v, 18.9 mL) at −100 °C was added dropwise *n*-BuLi (1.60 M solution in *n*-hexane, 1.05 mL, 1.68 mmol), and the resultant solution was stirred at −100 °C for 50 min. The reaction was quenched with saturated aqueous NH_4_Cl solution. The resultant mixture was extracted with EtOAc, and the organic layer was washed with brine, dried (Na_2_SO_4_), filtered, and concentrated under reduced pressure. The residue was roughly purified by flash column chromatography (silica gel, 5−10% EtOAc/hexanes) to give crude coupling product **25**, which was used in the next reaction without further purification: ^1^H NMR (600 MHz, C_6_D_6_) δ 8.04–8.01 (m, 3H), 7.91 (d, *J* = 8.3 Hz, 1H), 7.81–7.77 (m, 5H), 7.71 (s, 1H), 7.66–7.54 (m, 10H), 7.43 (d, *J* = 8.3 Hz, 1H), 7.33–7.30 (m, 2H), 7.25–7.15 (m, 10H), 6.70 (d, *J* = 8.3 Hz, 2H), 4.47–4.34 (m, 5H), 4.07 (m, 1H), 3.99 (m, 1H), 3.73 (dd, *J* = 9.7, 5.0 Hz, 1H), 3.64 (m, 1H), 3.58 (dd, *J* = 9.7, 6.4 Hz, 1H), 3.45 (dd, *J* = 9.7, 4.1 Hz, 1H), 3.43–3.40 (m, 2H), 3.27 (m, 1H), 3.04–2.98 (m, 3H), 2.68–2.61 (m, 2H), 2.23 (m, 1H), 2.13–2.08 (m, 2H), 2.04 (m, 1H), 1.93–1.81 (m, 4H), 1.78–1.52 (m, 10H), 1.48 (s, 3H), 1.29 (d, *J* = 6.9 Hz, 3H), 1.23–1.16 (m, 10H), 1.15 (d, *J* = 6.4 Hz, 3H), 0.99 (t, *J* = 7.8 Hz, 9H), 0.88 (d, *J* = 6.9 Hz, 1H), 0.58 (q, *J* = 7.8 Hz, 1H).

To a solution of the above coupling product **25** in CHCl_3_ (18 mL) at 0 °C was added TsOH·H_2_O (0.38 g, 2.0 mmol), and the resultant solution was stirred at 0 °C for 10.5 h. The reaction mixture was neutralized with Et_3_N, diluted with EtOAc, washed with brine, dried (Na_2_SO_4_), filtered, and concentrated under reduced pressure. Purification of the residue by flash column chromatography (silica gel, 2−5% EtOAc/hexanes) gave ketone **13** (1.45 g, 76% for the three steps) as a colorless oil: [α]_D_^24^ +38.2 (*c* 1.00, CHCl_3_); IR (film) 3853, 3838, 3566, 2930, 2361, 1716, 1684, 1558, 1507, 1457, 1080, 815 cm^−1^; ^1^H NMR (600 MHz, CDCl_3_) δ 7.82–7.70 (m, 16H), 7.47–7.33 (m, 15H), 4.74 (d, *J* = 11.9 Hz, 1H), 4.70–4.58 (m, 4H), 4.50 (d, *J* = 11.9 Hz, 1H), 4.27 (m, 1H), 3.80 (m, 1H), 3.64 (ddd, *J* = 9.1, 5.5, 3.2 Hz, 1H), 3.59–3.46 (m, 6H), 3.95 (dd, *J* = 9.6, 6.4 Hz, 1H), 3.38 (dd, *J* = 9.7, 4.1 Hz, 1H), 2.79 (dd, *J* = 16.9, 5.0 Hz, 1H), 2.42 (dd, *J* = 17.4, 16.9 Hz, 1H), 2.21 (m, 1H), 2.11 (ddd, *J* = 14.6, 7.3, 4.1 Hz, 1H), 1.84–1.60 (m, 15H), 1.22 (s, 3H), 1.02 (s, 9H), 0.94 (d, *J* = 6.4 Hz, 3H), 0.89 (d, *J* = 7.3 Hz, 3H), 0.84 (d, *J* = 6.4 Hz, 3H); ^13^C NMR (150 MHz, CDCl_3_) δ 210.6, 136.3, 136.1, 135.8, 135.61 (3C), 135.57 (3C), 134.0, 133.3, 133.2, 132.93, 132.89, 132.86, 129.5, 129.4, 128.2, 128.1, 127.9, 127.83 (2C), 127.80, 127.67, 127.65, 127.57, 127.53 (2C), 127.52 (2C), 126.4 (2C), 126.13, 126.11, 126.04, 125.99, 125.9, 125.84, 125.78, 125.7, 125.63, 125.60, 85.2, 82.6, 82.3, 81.3, 80.3, 74.9, 74.6, 73.0 (2C), 71.2, 71.0, 69.4, 68.344.1, 42.2, 41.2, 41.0, 39.8, 35.1, 33.0, 31.8, 31.1, 26.9 (3C), 26.6, 24.0, 22.6, 19.3, 18.5, 17.3, 13.9; HRMS (ESI) calcd for C_75_H_88_O_8_SiNa [(M + Na)^+^] 1167.6141, found 1167.6147.

**Alcohol 26.** To a solution of ketone **13** (1.18 g, 1.03 mmol) in MeOH/THF (1:1, v/v, 20 mL) at 0 °C was added NaBH_4_ (78.0 mg, 2.06 mmol), and the resultant solution was stirred at 0 °C for 40 min. The reaction was quenched with saturated aqueous NH_4_Cl solution. The resultant mixture was extracted with EtOAc, and the organic layer was washed with H_2_O and brine, dried (Na_2_SO_4_), filtered, and concentrated under reduced pressure. Purification of the residue by flash column chromatography (silica gel, 20% EtOAc/hexanes) gave alcohol **26** (1.14 g, 96%, d.r. >20:1) as a colorless clear oil: [α]_D_^25^ +21.1 (*c* 1.00, CHCl_3_); IR (film) 3853, 3839, 3734, 2931, 2362, 1698, 1558, 1541, 1457, 1319, 1150, 813, 742 cm^−1^; ^1^H NMR (600 MHz, CDCl_3_) δ 7.82–7.65 (m, 16H), 7.46–7.34 (m, 15H), 4.73–4.60 (m, 5H), 4.48 (d, *J* = 11.9 Hz, 1H), 4.28 (m, 1H), 3.81 (m, 1H), 3.59–3.49 (m, 5H), 3.41 (dd, *J* = 9.2, 4.1 Hz, 1H), 3.39 (dd, *J* = 9.2, 3.2 Hz, 1H), 3.36 (dd, *J* = 11.9, 4.6 Hz, 1H), 3.17 (ddd, *J* = 9.7, 9.6, 4.1 Hz, 1H), 3.06 (ddd, *J* = 11.5, 9.6, 4.6 Hz, 1H), 2.23 (m, 1H), 2.06 (ddd, *J* = 11.5, 8.2, 3.7 Hz, 1H), 2.01 (ddd, *J* = 11.9, 4.6, 4.6 Hz, 1H), 1.93 (m, 1H), 1.87 (ddd, *J* = 9.7, 9.6, 4.1 Hz, 1H), 1.76–1.58 (m, 12H), 1.55 (ddd, *J* = 11.9, 11.9, 11.4 Hz, 1H), 1.27 (m, 1H), 1.16 (dd, *J* = 14.1, 6.8 Hz, 1H), 1.11 (s, 3H), 1.04 (s, 9H), 0.97 (d, *J* = 6.9 Hz, 3H), 0.94 (d, *J* = 6.4 Hz, 3H), 0.89 (d, *J* = 7.3 Hz, 3H); ^13^C NMR (150 MHz, CDCl_3_) δ 136.5, 136.0, 135.9, 135.6 (3C), 134.00, 133.95, 133.3 (2C), 133.2, 132.91 (2C), 132.86, 129.48 (2C), 129.47 (2C), 128.1 (2C), 127.92, 127.86, 127.82 (2C), 127.80 (2C), 127.7 (2C), 127.6, 127.5 (3C), 126.4, 126.3, 126.2, 126.17, 126.13, 126.08, 126.02, 125.8 (2C), 125.7, 125.63, 125.57, 85.1, 82.4, 81.6, 81.4, 74.6, 74.5, 73.2, 73.0, 72.1, 71.2, 70.8, 70.0, 68.3, 43.3, 42.2, 41.1, 40.0, 36.1, 35.2, 33.1, 31.3, 31.1, 26.9 (3C), 24.3, 19.2, 18.9, 17.1, 15.0, 14.0; HRMS (ESI) calcd for C_75_H_90_O_8_SiNa [(M + Na)^+^] 1169.6297, found 1169.6288.

**Diol 27.** To a solution of alcohol **26** (1.14 g, 0.993 mmol) in THF (15 mL) at 0 °C was added TBAF (1.0 M solution in THF, 2.0 mL, 2.0 mmol), and the resultant solution was stirred at room temperature for 2.5 h. The reaction was quenched with saturated aqueous NH_4_Cl solution. The resultant mixture was extracted with EtOAc, and the organic layer was washed with H_2_O and brine, dried (Na_2_SO_4_), filtered, and concentrated under reduced pressure. Purification of the residue by flash column chromatography (silica gel, 30−80% EtOAc/hexanes) gave diol **27** (0.83 g, 92%) as a colorless amorphous solid: [α]_D_^24^ +32.6 (*c* 1.00, CHCl_3_); IR (film) 3853, 3648, 3420, 2932, 2362, 1716, 1540, 1508, 1457, 1374, 1071, 815, 772 cm^−1^; ^1^H NMR (600 MHz, CDCl_3_) δ 7.82–7.71 (m, 12H), 7.47–7.37 (m, 9H), 4.73–4.59 (m, 5H), 4.45 (d, *J* = 11.5 Hz, 1H), 4.26 (m, 1H), 3.79 (m, 1H), 3.59–3.48 (m, 5H), 3.42 (dd, *J* = 9.2, 4.1 Hz, 1H), 3.32 (m, 1H), 3.28 (ddd, *J* = 10.1, 9.7, 4.1 Hz, 1H), 3.22 (m, 1H), 3.12 (ddd, *J* = 9.7, 9.6, 4.6 Hz, 1H), 2.22 (m, 1H), 2.07 (ddd, *J* = 11.5, 6.4, 3.2 Hz, 1H), 2.01 (ddd, *J* = 11.9, 4.6, 4.6 Hz, 1H), 1.87–1.52 (m, 17H), 1.27 (m, 1H), 1.15 (s, 3H), 0.92 (d, *J* = 6.4 Hz, 3H), 0.89 (d, *J* = 7.4 Hz, 3H), 0.87 (d, *J* = 6.9 Hz, 3H); ^13^C NMR (150 MHz, CDCl_3_) δ 136.4, 136.0, 135.8, 134.8, 133.2 (2C), 133.2, 132.9 (2C), 132.9, 128.13, 128.10, 127.92, 127.86, 127.83, 127.81, 127.7 (2C), 127.6, 126.5, 126.4, 126.2, 126.1, 126.06, 126.04, 125.82 (2C), 125.77, 125.63, 125.60, 85.0, 82.0, 81.8, 80.7, 77.0, 74.5, 74.6, 73.1, 73.0, 71.2, 70.9, 68.8, 68.7, 68.3, 60.4, 44.2, 42.1, 41.1, 39.9, 36.1, 35.1, 33.1, 31.2, 30.7, 26.6, 24.0, 19.7, 17.0, 14.0; HRMS (ESI) calcd for C_59_H_72_O_8_Na [(M + Na)^+^] 931.5119, found 931.5122.

**Lactone 14.** To a solution of diol **27** (0.83 g, 0.91 mmol) in CH_2_Cl_2_ (2.5 mL) at room temperature were added PhI(OAc)_2_ (0.64 g, 2.0 mmol) and TEMPO (14 mg, 0.091 mmol), and the resultant solution was stirred at room temperature for 15 h 10 min. The reaction was quenched with a 1:1 mixture of saturated aqueous Na_2_SO_3_ solution and saturated aqueous NaHCO_3_ solution. The resultant mixture was diluted with EtOAc, washed with H_2_O and brine, dried (Na_2_SO_4_), filtered, and concentrated under reduced pressure. Purification of the residue by flash column chromatography (silica gel, 20−30% EtOAc/hexanes) gave lactone **14** (0.76 g, 92%) as a colorless oil: [α]_D_^24^ +51.8 (*c* 1.00, CHCl_3_); IR (film) 3734, 3648, 2934, 2362, 1740, 1540, 1508, 1457, 1375, 1063, 816 cm^−1^; ^1^H NMR (600 MHz, CDCl_3_) δ 7.82–7.70 (m, 11H), 7.46–7.39 (m, 10H), 4.07–4.60 (m, 5H), 4.47 (d, *J* = 11.9 Hz, 1H), 4.23 (m, 1H), 3.91 (dd, *J* = 12.4, 4.1 Hz, 1H), 3.79–3.74 (m, 2H), 3.61–3.50 (m, 3H), 3.41–3.37 (m, 2H), 3.30 (ddd, *J* = 11.0, 9.7, 4.6 Hz, 1H), 2.71 (m, 1H), 2.23–2.18 (m, 2H), 2.08–2.03 (m, 2H), 1.87–1.58 (m, 11H), 1.51 (dd, *J* = 11.9, 11.0 Hz, 1H), 1.33 (d, *J* = 7.4 Hz, 3H), 1.30–1.23 (m, 5H), 0.95 (d, *J* = 6.4 Hz, 3H), 0.88 (d, *J* = 6.9 Hz, 3H); ^13^C NMR (150 MHz, CDCl_3_) δ 136.4, 136.2, 135.7, 133.3 (2C), 133.2, 132.94, 132.89, 132.86, 128.3, 128.15, 128.06, 127.96, 127.83 (2C), 127.79, 127.68, 127.66, 127.59, 126.4, 126.3, 126.14, 126.11, 126.0, 125.9 (2C), 125.8, 126.7, 125.6 (2C), 85.2, 81.8, 81.4, 79.0, 78.2, 76.9, 74.8, 74.6, 73.2, 73.0, 71.4, 70.8, 70.5, 68.3, 43.0, 42.2, 41.2, 40.0, 35.1, 34.6, 33.1, 32.3, 31.1, 26.3, 23.2, 19.2, 17.2, 14.4, 13.9; HRMS (ESI) calcd for C_59_H_68_O_8_Na [(M + Na)^+^] 927.4806, found 927.4827.

**Diene 15.** To a solution of lactone **14** (0.39 g, 0.43 mmol) and PhNTf_2_ (338 mg, 0.946 mmol) in THF/HMPA (20:1, v/v, 12.6 mL) at −78 °C was added KHMDS (0.5 M solution in toluene, 1.7 mL, 0.85 mmol), and the resultant solution was stirred at −78 °C for 50 min. The reaction mixture was allowed to warm to room temperature and then concentrated under reduced pressure. The residue was extracted with hexanes, and the extract was concentrated under reduced pressure to give crude enol triflate **29**, which was immediately used in the next reaction without further purification.

To a suspension of the above enol triflate, LiCl (109 mg, 2.58 mmol), and Pd(PPh_3_)_4_ (49.7 mg, 0.0427 mmol) in 1,4-dioxane (10 mL) was added vinyl tri-*n*-butyltin (0.37 mL, 1.3 mmol), and the resultant mixture was heated at 80 °C for 80 min. The reaction mixture was concentrated under reduced pressure, and the residue was purified by flash column chromatography (10 wt% K_2_CO_3_/silica gel, 20−100% EtOAc/hexanes) to give diene **15**. To remove traces of organotin byproducts, this material was taken up in 20% aqueous KF solution/THF (1:1, v/v, 16 mL) and stirred vigorously at room temperature for 35 min. The resultant mixture was extracted with EtOAc, and the organic layer was washed with H_2_O and brine, dried (Na_2_SO_4_), filtered, and concentrated under reduced pressure. The residue was passed through a pad of 10 wt% K_2_CO_3_/silica gel (10−20% EtOAc/hexanes), and the fractions containing **15** were collected and concentrated under reduced pressure. To remove traces of palladium salts, the residue was taken up in EtOAc/saturated aqueous NaHCO_3_ solution (1:1, v/v, 16 mL), treated with DL-serine (903 mg), and stirred vigorously at room temperature. The resultant mixture was extracted with EtOAc, and the organic layer was washed with H_2_O and brine, dried (Na_2_SO_4_), filtered, and concentrated under reduced pressure. Purification of the residue by flash column chromatography (10 wt% K_2_CO_3_/silica gel, 15−20% EtOAc/hexanes) gave diene **15** (249 mg, 63% for the two steps) as a colorless oil: [α]_D_^24^ +92.3 (*c* 1.00, benzene); IR (film) 3853, 3734, 3648, 2362, 1716, 1541, 1507, 1457, 1081, 772 cm^−1^; ^1^H NMR (600 MHz, C_6_D_6_) δ 7.81 (s, 1H), 7.72 (s, 1H), 7.69–7.60 (m, 10H), 7.52 (d, *J* = 8.3 Hz, 1H), 7.44 (d, *J* = 8.2 Hz, 1H), 7.42 (d, *J* = 8.2 Hz, 1H), 7.28–7.20 (m, 6H), 6.42 (dd, *J* = 16.9, 12.4 Hz, 1H), 5.83 (d, *J* = 16.9 Hz, 1H), 5.05 (d, *J* = 12.4 Hz, 1H), 4.78 (d, *J* = 11.9 Hz, 1H), 4.68 (d, *J* = 13.6 Hz, 1H), 4.51–4.45 (m, 4H), 4.26 (d, *J* = 11.9 Hz, 1H), 4.14 (m, 1H), 3.92 (ddd, *J* = 9.6, 4.6, 4.1 Hz, 1H), 3.52–3.39 (m, 7H), 2.46 (ddd, *J* = 11.9, 4.6, 4.1 Hz, 1H), 2.30 (d, *J* = 16.5 Hz, 1H), 2.16–1.74 (m, 10H), 1.65–1.52 (m, 3H), 1.46 (s, 3H), 1.30–1.52 (m, 8H), 0.92–0.84 (m, 4H); ^13^C NMR (150 MHz, C_6_D_6_) δ 144.9, 137.5, 137.0, 136.6, 133.94, 133.91, 133.85, 133.5, 133.4, 128.3 (2C), 128.2, 128.1, 128.0 (2C), 127.96, 127.91 (2C), 127.80, 127.7, 126.6, 126.5, 126.4 (2C), 126.29, 126.27, 126.1, 126.1, 126.0, 125.9, 125.8, 125.7, 112.7, 108.1, 85.4, 82.9, 82.1, 81.2, 75.8, 75.5, 75.0, 73.3, 73.1, 71.5, 71.0, 70.6, 68.3, 44.9, 42.7, 41.5, 40.6, 35.5, 33.6, 33.1, 31.6, 27.4, 24.2, 17.6, 17.2, 15.6, 14.1; HRMS (ESI) calcd for C_61_H_70_O_7_Na [(M + Na)^+^] 937.5014, found 937.5015.

**Alcohol 31.** To a solution of diene **15** (16.9 mg, 0.0185 mmol) in CH_2_Cl_2_ (1.2 mL) at −78 °C was added DMDO (ca. 0.08 M solution in acetone, 0.55 mL, 0.44 mmol), and the resultant solution was allowed to warm to −40 °C over a period of 30 min and further stirred at −40 °C for 20 min. The reaction was quenched with saturated aqueous Na_2_SO_3_ solution. The resultant mixture was extracted with EtOAc, and the organic layer was washed with H_2_O and brine, dried (Na_2_SO_4_), filtered, and concentrated under reduced pressure to give epoxide **30**, which was immediately used in the next reaction without further purification: ^1^H NMR (600 MHz, C_6_D_6_) δ 7.88 (s, 1H), 7.83 (s, 1H), 7.78–7.69 (m, 10H), 7.60 (d, *J* = 8.3 Hz, 1H), 7.55 (d, *J* = 8.2 Hz, 1H), 7.50 (d, *J* = 8.2 Hz, 1H), 7.37–7.30 (m, 6H), 5.89 (dd, *J* = 17.4, 10.6 Hz, 1H), 5.76 (d, *J* = 17.4 Hz, 1H), 5.26 (d, *J* = 10.6 Hz, 1H), 4.85 (d, *J* = 11.9 Hz, 1H), 4.75 (d, *J* = 11.9 Hz, 1H), 4.61–4.55 (m, 4H), 4.34 (d, *J* = 11.9 Hz, 1H), 4.19 (m, 1H), 4.00 (dd, *J* = 10.6, 4.6 Hz, 1H), 3.95 (m, 1H), 3.58–3.46 (m, 5H), 3.42 (ddd, *J* = 9.6, 4.6, 4.6 Hz, 1H), 2.39 (ddd, *J* = 12.4, 4.6, 4.6 Hz, 1H), 2.37 (d, *J* = 12.8 Hz, 1H), 2.19 (ddd, *J* = 12.8, 11.5, 11.0 Hz, 1H), 2.13–2.08 (m, 2H), 2.02–1.69 (m, 9H), 1.63 (ddd, *J* = 11.0, 6.9, 6.8 Hz, 1H), 1.61 (m, 1H), 1.34 (m, 1H), 1.32 (d, *J* = 6.4 Hz, 3H), 1.21 (s, 3H), 1.06 (s, 3H), 0.97 (d, *J* = 6.9 Hz, 3H).

To a solution of the above epoxide **30** in THF (1.2 mL) at −78 °C was added dropwise DIBALH (1.02 M solution in *n*-hexane, 0.040 mL, 0.041 mmol), and the resultant solution was allowed to warm to −40 °C over a period of 30 min and further stirred at −40 °C for 35 min. The reaction was quenched with MeOH. The resultant solution was diluted with EtOAc and saturated aqueous potassium sodium tartrate solution and stirred vigorously at room temperature overnight. The resultant mixture was extracted with EtOAc, and the organic layer was washed with brine, dried (Na_2_SO_4_), filtered, and concentrated under reduced pressure. Purification of the residue by flash column chromatography (silica gel, 20−30% EtOAc/hexanes) gave alcohol **31** (14.8 mg, 86% for the two steps) as a colorless oil: ^1^H NMR (600 MHz, CDCl_3_) δ 7.82–7.70 (m, 12H), 7.47–7.38 (m, 9H), 5.83 (ddd, *J* = 17.5, 11.0, 6.5 Hz, 1H), 5.31 (m, 1H), 5.20 (m, 1H), 4.71–4.59 (m, 5H), 4.47 (d, *J* = 11.5 Hz, 1H), 4.25 (m, 1H), 3.78 (m, 1H), 3.70–3.66 (m, 2H), 3.60–3.47 (m, 3H), 3.45–3.36 (m, 2H), 3.28 (ddd, *J* = 11.0, 11.0, 5.0 Hz, 1H), 3.06 (dd, *J* = 12.0, 3.0 Hz, 1H), 2.21 (m, 1H), 2.16–2.06 (m, 2H), 2.02 (d, *J* = 12.0 Hz, 1H), 1.90–1.82 (m, 2H), 1.75–1.59 (m, 11H), 1.34 (br s, 1H), 1.28–1.23 (m, 7H), 0.95 (d, *J* = 6.5 Hz, 3H), 0.87 (d, *J* = 6.4 Hz, 3H). The ^1^H NMR data matched those previously reported (Ishigai et al., 2013).

**A/BCD-ring fragment 2.** To a solution of alcohol **31** (60.3 mg, 0.0646 mmol) and 2,6-lutidine (0.040 mL, 0.34 mmol) in CH_2_Cl_2_ (7.0 mL) at 0 °C was added TMSOTf (0.025 mL, 0.14 mmol), and the resultant solution was stirred at 0 °C for 30 min. The reaction mixture was diluted with EtOAc, washed with saturated aqueous NaHCO_3_ solution and brine, dried (Na_2_SO_4_), filtered, and concentrated under reduced pressure. Purification of the residue by flash column chromatography (silica gel, 5% Et_2_O/benzene) gave the A/BCD-ring fragment **2** (58.9 mg, 91%) as a colorless oil: ^1^H NMR (600 MHz, CDCl_3_) δ 7.82–7.72 (m, 12H), 7.46–7.39 (m, 9H), 5.84 (ddd, *J* = 17.4, 11.0, 4.0 Hz, 1H), 5.26 (d, *J* = 17.4 Hz, 1H), 5.11 (d, *J* = 11.0 Hz, 1H), 4.72–4.60 (m, 5H), 4.47 (d, *J* = 12.0 Hz, 1H), 4.26 (m, 1H), 3.79 (m, 1H), 3.72–3.66 (m, 2H), 3.60–3.48 (m, 3H), 3.43–3.36 (m, 2H), 3.27 (ddd, *J* = 10.5, 10.5, 5.0 Hz, 1H), 3.09 (dd, *J* = 13.0, 3.5 Hz, 1H), 2.20 (m, 1H), 2.14–2.06 (m, 2H), 2.04 (d, *J* = 12.5 Hz, 1H), 1.89–1.56 (m, 13H), 1.28–1.19 (m, 7H), 0.95 (d, *J* = 6.5 Hz, 3H), 0.87 (d, *J* = 7.0 Hz, 3H), 0.08 (s, 9H). The ^1^H NMR data matched those previously reported (Ishigai et al., 2013).

1. **References**

Ishigai, K., Fuwa, H., Hashizume, K., Fukazawa, R., Cho, Y., Yotsu-Yamashita, M., and Sasaki, M. (2013). Total synthesis and biological evaluation of (+)-gambieric acid A and its analogues. *Chem. Eur. J.* 19, 5276–5288.
